# Quantification of Salicylates and Flavonoids in Poplar Bark and Leaves Based on IR, NIR, and Raman Spectra

**DOI:** 10.3390/molecules27123954

**Published:** 2022-06-20

**Authors:** Sylwester Mazurek, Maciej Włodarczyk, Sonia Pielorz, Piotr Okińczyc, Piotr M. Kuś, Gabriela Długosz, Diana Vidal-Yañez, Roman Szostak

**Affiliations:** 1 Department of Chemistry, University of Wrocław, 14 F. Joliot-Curie, 50-383 Wrocław, Poland; sonia.pielorz@chem.uni.wroc.pl (S.P.); roman.szostak@chem.uni.wroc.pl (R.S.); 2 Department of Pharmacognosy and Herbal Medicines, Faculty of Pharmacy, Wroclaw Medical University, 211a Borowska, 50-556 Wrocław, Poland; piotr.okinczyc@umw.edu.pl (P.O.); piotr.kus@umw.edu.pl (P.M.K.); gabrysia_d@interia.pl (G.D.); diana.vidal253@gmail.com (D.V.-Y.); 3 Faculty of Pharmacy, University of Barcelona, Joan XXIII, 27-31, 08014 Barcelona, Spain

**Keywords:** poplar, salicylates, flavonoids, multivariate analysis, vibrational spectroscopy

## Abstract

Poplar bark and leaves can be an attractive source of salicylates and other biologically active compounds used in medicine. However, the biochemical variability of poplar material requires a standardization prior to processing. The official analytical protocols used in the pharmaceutical industry rely on the extraction of active compounds, which makes their determination long and costly. An analysis of plant materials in their native state can be performed using vibrational spectroscopy. This paper presents for the first time a comparison of diffuse reflectance in the near- and mid-infrared regions, attenuated total reflection, and Raman spectroscopy used for the simultaneous determination of salicylates and flavonoids in poplar bark and leaves. Based on 185 spectra of various poplar species and hybrid powdered samples, partial least squares regression models, characterized by the relative standard errors of prediction in the 4.5–9.9% range for both calibration and validation sets, were developed. These models allow for fast and precise quantification of the studied active compounds in poplar bark and leaves without any chemical sample treatment.

## 1. Introduction

**Figure 1:**
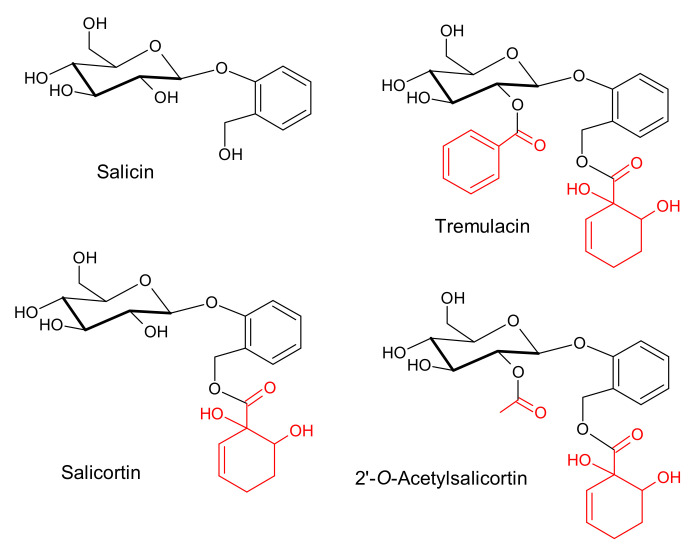
Molecular structure of salicin and its derivatives; salicin core (black) and structural modifications in the dominant derivatives are highlighted in red.

The characteristic constituents of poplar (*Populus* L.) bark, leaves, and buds, as for the entire willow family (Salicaceae), are derivatives of salicin (Figure 1). These metabolites play an important role in plant defenses against herbivores and act as anti-inflammatory and anticoagulant agents in the human body [1]. A metabolic salicylate grid exists in willow family plants, in which salicylates might be converted with salicin as an intermediate. At the same time, their total concentration remains unchanged [2]. Apart from salicylates, polyphenol compounds are present in Salicaceae bark and leaves; among them, tannins, flavanones, and flavonols are the most abundant [3,4,5,6,7,8].

The specific biological activity of poplar-derived raw herbal drugs is a result of salicylates and flavonoids action [1]. The growing conditions of poplar trees and the preparation and processing methods of plant materials can influence the final contents of active compounds [9]. The concentration of active compounds varies between poplar species and hybrids. In bark, salicylates are usually present in the 0.5–10% range, and in leaves, they can reach 15% [10]. The concentration of flavonoids is approximately an order of magnitude lower, and tannins can constitute more than 10% of bark [11].

The variability of plant materials’ chemical compositions is a basic limitation preventing the direct utilization of raw materials in the pharmaceutical, cosmetic, and food industries. In the case of herbal medicine production, it is critically important to monitor the contents of the active compounds in different batches. Therefore, a standardization of the used material is required to ensure the appropriate quality of preparations obtained from plant tissues. The official methods of an active ingredient analysis in plant materials are usually time-consuming, labor-intensive, and generate chemical waste. Determining any single analytical marker or a group of markers requires separate protocols, measuring equipment, and reagent kits, including standards. Additionally, various protocols result in different extraction yields, which makes the comparison of different methods problematic.

Among the analytical methods providing chemical data without such difficulties, particular attention should be paid to vibrational spectroscopy techniques. The infrared (IR) and Raman spectra provide qualitative and quantitative information on the analyzed material, often without any sample treatment. There is no need to dissolve the sample and extract the compounds of interest, which significantly simplifies the analysis and shortens the time. This is particularly important in the case of a large sample set analysis. The bands in the IR and Raman spectra, resulting from an excitation beam’s interaction with the structural moieties of the molecules present in the sample, besides structural information, allow linkage of the signal intensity with the number of excited oscillators, enabling their quantification. Bands originating from various compounds strongly overlap, making vibrational spectra of the natural products complex. However, multivariate data treatment techniques facilitate detailed analyses of different compound groups, including active substances and nutrients based on the vibrational spectra of the plant material [12,13].

Near-infrared spectroscopy is the most frequently used vibrational technique in plant material analysis. Transmittance and reflectance near-infrared (NIR) spectra were utilized to study the physicochemical properties of foliage samples, including the determination of different organic constituents; chlorophyll and carotenoid contents; and elemental analyses of carbon, nitrogen, and phosphorus, as well as the ^13^C and ^15^N levels [14,15,16,17,18]. NIR spectroscopy was applied to analyze the phenolic compounds and alkaloids in green tea leaves, flavonoids in rice grains, and tannins in greater lotus samples [19,20,21,22]. Changes in the chemical compositions of plant materials detected by the NIR technique enabled field phenotyping of the leaf samples and the classification of wood and bark samples [23,24]. In the case of poplar material analysis, NIR data were used to quantify tannins and phenolic glycosides in leaf samples [5]. Furthermore, research has reported an application of this technique for salicinoids determination in the fresh and dry leaves of aspen and paper birch [25].

The attenuated total reflection (ATR) spectra in the mid-infrared (MIR) region supported the classification of the heartwood samples of pine species and was used to track the polysaccharide content as grapes ripened [26,27]. There have been several analyses of betulin and betulinic acid, potential anticancer drugs and HIV inhibitor candidates, in birch bark based on ATR and transmission IR spectra [28,29,30,31]. Diffuse reflectance (DRIFTS) IR spectroscopy was used to quantify rosmarinic acid in Lamiaceae herbs and leaf pigment analyses [32,33,34]; it was also used to detect saffron adulteration and for genotyping barley samples [35,36].

Raman spectroscopy allowed the analysis of cellulose, lignocellulosic compounds, and polyphenolic compound discrimination in bark samples [37,38]. Based on Raman spectra, quinine in cinchona bark, quercetin in alcohol extracts from onion peels, aspalathin in green rooibos, and pigments of tea leaves were quantified [39,40,41,42]. Raman spectra were also utilized in triterpene compound analyses in birch bark [28,29,30]. This technique was also used to model the lignin content in bark for poplar sample genotyping [43].

In our previous studies, we demonstrated the advantages of vibrational spectroscopy techniques in the analyses of natural products. IR and Raman spectra of an untreated material enabled the quantification of active compounds in *Potentilla tormentilla* rhizomes and the determination of the total polyphenol content and nutritional parameters of bee pollen samples [44,45]. In this study, we compare an application of Fourier-transform (FT) Raman, DRIFTS in the MIR and NIR regions, and ATR methods in the modeling of salicylates and flavonoids contents in bark and leaves from various poplar species and hybrids.

## 2. Results and Discussion

### 2.1. Vibrational Spectra of Poplar Bark and Leaves

**Figure 2:**
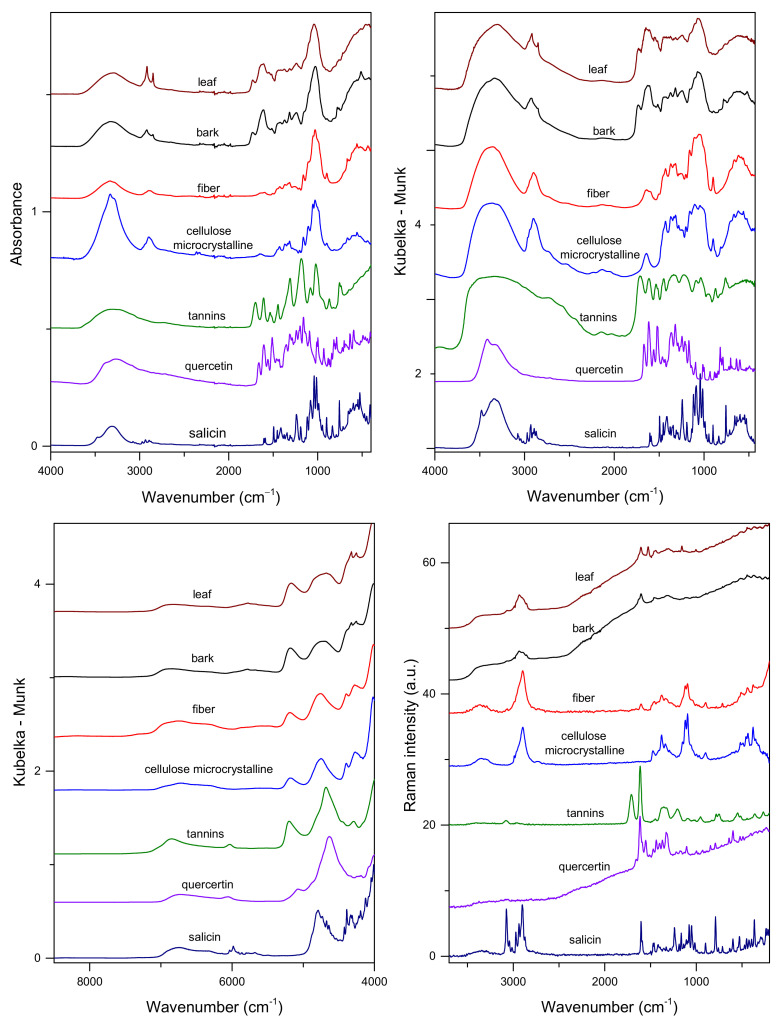
Vibrational spectra of poplar bark, leaves, and reference compounds: top panel—ATR (**left**) and DRIFTS (**right**) and bottom panel—NIR (**left**) and Raman (**right**).

**Figure 3:**
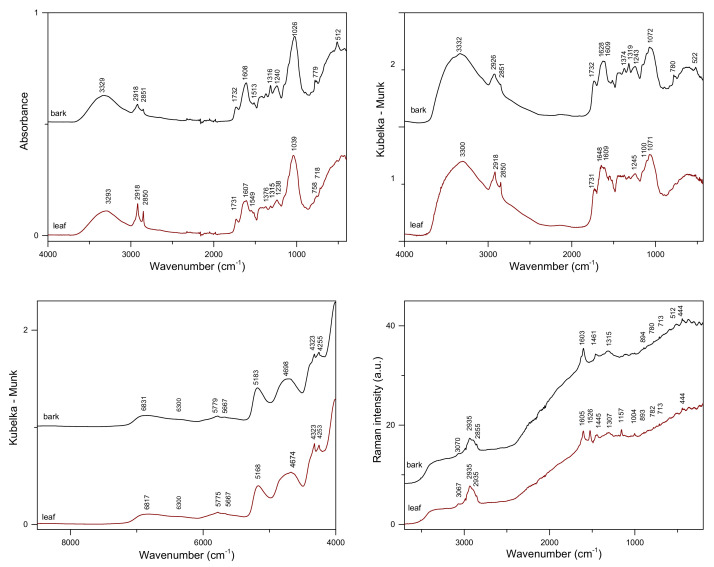
Vibrational spectra of poplar bark and leaves: top panel—ATR (**left**) and DRIFTS (**right**) and bottom panel—NIR (**left**) and Raman (**right**).

Figure 2 and Figure 3 show the average vibrational spectra of the studied samples. Polysaccharides constitute the primary structural material in bark and leaves; thus, it is expected that the contribution of carbohydrates in the obtained spectra will be dominant. However, differences in their relative contents and the presence of other substances make the spectra of bark and leaves slightly different. Besides cellulose, hemicellulose, and pectin, bark samples contain lignin, high-molecular-weight tannins, and suberin. For leaves, protein, C16/C18 polyesters of waxes and cutin, chlorophylls, and carotenoids can be listed as additional metabolite groups.

In the ATR and DRIFTS/MIR spectra, the most intense bands originating from ν(C-O) and ν(C–O–C) vibrations of polysaccharide skeletons are visible in the 900–1185 cm^−1^ range [13]. These broad bands, with the maxima at 1026/1039 cm^−1^ and 1072/1071 cm^−1^ for the bark/leaf samples, dominate the ATR and DRIFTS spectra, respectively. Another strong massif visible in the 1500–1700 cm^−1^ range can be assigned to the characteristic vibrations of polyphenolic compounds [13,44]. In this spectral region, additional contributions from protein vibrations (Amide I band) can significantly influence the contour of the IR spectra of leaves. In the C–H stretching region, various groups of chemical compounds can contribute; especially, signals of esters of aliphatic fatty acids and polysaccharides are easily recognizable [13,44]. Both bands, the ν_s_(C–H) at about 2850 cm^−1^ and the ν_as_(C–H) at 2918, are visible in the ATR and DRIFTS spectra, with a noticeably higher intensity in the leaves’ spectra. In the case of the DRIFTS spectrum of bark, the ν_as_(C–H) band is located at 2926 cm^−1^.

NIR spectra of the studied tissues are less specific in comparison to MIR spectra. Overtones of fundamental vibrations and their combinations form in the near-IR range broad, overlapped bands. An intense peak with a maximum at 4698/4674 cm^−1^ in poplar bark/leaves is commonly associated with the CH group absorption of aromatic species, particularly those of a phenolic nature [22]. The broad signal in the 4900–5400 cm^−1^ range, with the maxima at 5183/5168 cm^−1^, can be linked to the presence of polysaccharides (Figure 2). Two separate bands, with the maxima at about 4252 and 4323 cm^−1^, originating from δ(C–H) and ν(C–H) combinations of aliphatic fatty acid vibrations, can be distinguished [46]. Other, less intense bands in the 6100–7500 cm^−1^ range correspond to the presence of carbohydrates. The band with a maximum at 6831/6817 cm^−1^ can be assigned to polysaccharides, and the band with a maximum at approximately 6300 cm^−1^ is assigned to cellulose. In the NIR spectra of poplar bark, a weak signal, which can be assigned to the presence of lignin, occurs at 5980 and 5775 cm^−1^ [47].

In spite of the high polysaccharide content in the studied samples, their spectral contributions in the low wavenumber range of the Raman spectra of bark and leaves are very weak. Only a broad band in the 2800–3000 cm^−1^ range, corresponding to ν(C–H) vibrations, is visible [44,45]. A very characteristic intense band in the Raman spectra of the two analyzed tissues can be found at approximately 1604 cm^−1^. It corresponds to the C=C vibrations of an aromatic moiety [13,37]. This contribution is present in the spectra of sali-cylates and flavonoids (Figure 2). Additional intense bands with the maxima at 1526, 1157, and 1004 cm^−1^, originating from the ν(C=C), ν(C–C), and δ(C=CH) vibrations of carotenoids [48], respectively, are present in the spectra of leaves. The last two mentioned contributions in the Raman spectra overlap the ν(CO), ν(CC), ν(CCO), and *δ*(OCH) shares of cellulose, pectin, and lignin, recognizable in the Raman spectrum of a leaf [13,38]. Notably, the use of an excitation laser beam with a wavelength outside the visible range (λ = 1064 nm) did not prevent fluorescence when recording the Raman spectra of poplar samples. This phenomenon masks weaker Raman features originating from compounds present in the analyzed tissues.

**Figure 4:**
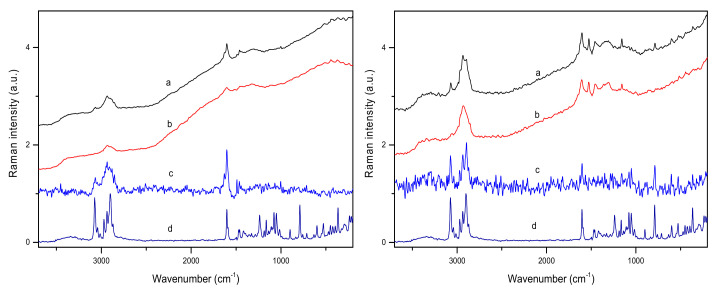
Raman spectra of poplar bark (**left**) and leaves (**right**) containing the maximal (a) and minimal (b) contents of salicylates, difference spectra (c), and the spectrum of salicin (d).

Our studies focused on the analysis of two groups of active compounds in poplar bark and leaf samples, namely salicylates and flavonoids. The salicylates content in the analyzed samples reached 10% (*w*/*w*). It would be interesting to check whether it is possible to distinguish samples containing the highest concentration of these active compounds directly by only comparing the spectra. As the plots in Figure 2 suggest, it can be realized using Raman spectra. However, IR techniques could also support such a selection, as the features in the difference spectra seem to be linked mainly to the presence of salicylates. As previously noted, apart from salicin, other salicylates and phenolic glycosides, which may contribute to the same regions of vibrational spectra, are present in the analyzed materials. Figure 4 shows Raman spectra obtained for samples containing extreme TSA concentrations in the leaves and bark samples, and Figure S1 in the Supplementary Materials presents ATR spectra for these tissues.

### 2.2. Principal Component Analysis (PCA)

The studied samples’ diversity relates to differences in the chemical compositions of plant tissues. It manifests itself as a change in the relative contents of active compounds. As the vibrational spectra of the studied samples are complex (Figure 3), their straightforward analysis is not an easy task, but it can be supported by the use of chemometric techniques. By applying PCA decomposition and utilizing the loading plots with a spectrum-like representation, it is possible to identify the spectral contributions of different chemical groups that are present in the studied material. Additionally, PCA score plots can be used to discriminate samples according to their chemical compositions. However, the sample distributions in the PC1/PC*n* (*n* = 2, 3, …) coordination systems can vary, moving from one spectroscopic technique to another. The spectral variance in an analyzed data sets is related to the compounds’ spectral contributions and the interactions of electromagnetic radiation with the samples, which are characteristic for the applied experimental technique.

**Figure 5:**
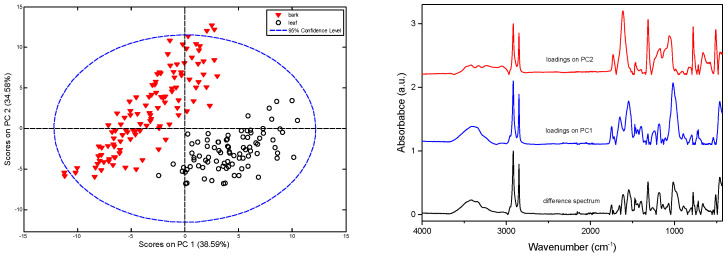
PCA for ATR spectra of poplar bark and leaves for merged data sets: **left**—score plots and **right**—plots of the absolute values of the PC1 and PC2 loadings and subtraction result between the average leaf and bark spectra.

Despite the superficial resemblance of the average spectra of bark and leaves, the chemical composition differences of these two tissues resulted in clearly separated groups of points in the PCA score plots for the model based on the merged set of data (Figure 5). Therefore, a detailed PCA analysis was performed separately for the two material types studied.

**Figure 6:**
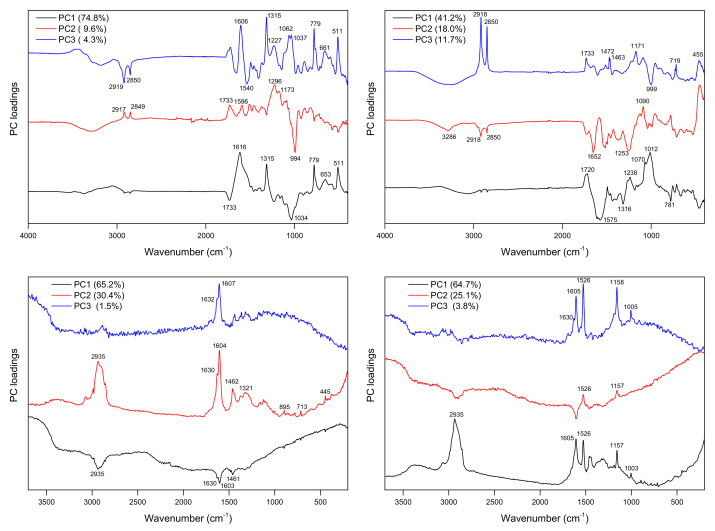
PCA loadings for poplar bark (**left**) and leaves (**right**) calculated on the basis of ATR (**top**) and Raman (**bottom**) spectra.

Figure 6 shows the obtained PCA loadings for ATR and Raman data, while these plots for DRIFTS and NIR data are presented in Figure S2 in the Supplementary Materials. These plots distinguish spectral variations originating from structurally related substances.

PCA decomposition of the MIR spectra of poplar bark samples revealed a set of characteristic signals with the maxima at 1615, 1315, 779, and 511 cm^−1^, which appear in the loadings of ATR (PC1 and PC3) and DRIFTS (PC2) plots, indicating pronounced variations of polyphenolic glycoside contents in the studied materials. In the loadings plots in the range of carbohydrate ν(C-O-C) vibrations, there are visible contributions, with the maxima at 1034 cm^−1^ (PC1), 1062 cm^−1^ (PC3), and 1091 and 1112 cm^−1^ (PC2) for ATR data and at 1066, 1112 and 1163 cm^−1^ (PC1), and 1170 cm^−1^ (PC2 and PC3) for DRIFTS data. The features characteristic for fatty acids can be found in the PC2 and PC3 loadings for ATR data (i.e., signals with the maxima at about 1733, 2850, and 2919 cm^−1^), but an analysis of the DRIFTS spectra revealed a strong band of pectin ν(C=O) vibration (PC2 loadings). For the PCA of Raman data after multipoint baseline correction, the signals of salicylates and flavonoids, both clearly visible in the 1580–1650 cm^−1^ range, dominate PC1 and PC2 loadings, but shares originating from other compound groups can be found in the 1000–1500 cm^−1^ range. The PCA of Raman spectra without baseline subtraction discriminates bark samples according to the fluorescence (Figure S3 in the Supplementary Materials), which is probably linked to the content of the lignocellulosic compound biomass.

Several characteristic features can also be found in the loading plots obtained by PCA decomposition of spectral matrices for leaves. For example, PCA for Raman data reveals the variance related to the presence of chlorophylls and carotenoids. The latter group of compounds shows significant contributions in the loading plots. A high Raman cross-section typical for compounds contained in their structure polyene chain makes carotenoids easily recognizable in the spectra, even when their content in the analyzed tissue is relatively low. The indicative bands of carotenoids with the maxima at about 1526, 1157, and 1004 cm^−1^ of the ν(C=C), ν(C-C), and δ(C=CH) vibrations, respectively, can be detected in the Raman spectra of poplar leaves and plots of the PC1 and PC3 loadings. Figure S4 in the Supplementary Materials shows an overlap of these loadings with the beta-carotene spectrum. Additional spectral features can also be detected by PCA on the ATR and DRIFTS spectra of leaves. The signals with the maxima at 3287 and 1650 cm^−1^ found in PC1/PC2 loading plots can be assigned to the ν(N-H) and Amide I contributions of proteins. Notably, the PC1/PC2 scores plot obtained from the ATR spectra PCA of leaves also allowed for separating samples in accordance with the salicylate content (Figure S5 in the Supplementary Materials).

### 2.3. PLS Quantification of Active Compounds in Poplar Material

To construct quantitative calibration models for the two studied groups of active compounds, vibrational spectra of the poplar bark and leaves were combined with the reference analyses for TSA and TFL performed for each sample according to the UHPLC–UV and UV–Vis protocols, respectively, as described in Section 3.2. Calibration models were constructed separately for each type of plant material and spectroscopic technique used.

#### 2.3.1. Determination of Total Salicylates (TSA)

In the studied samples, the salicylate content varied by 0.6–8.1% and 0.5–12.0% (*w*/*w*) for the bark and leaves, respectively. However, in more than 85% of the bark samples, the TSA concentration was found to be 1–4% (*w*/*w*), and only a few samples contained a higher level of salicylates. Figure 3 and Figure S1 in the Supplementary Materials show a comparison of the Raman spectra for poplar bark and leaf samples containing extreme TSA concentrations. PLS models were built by applying the characteristic spectral ranges for salicylates. Nevertheless, construction of the most efficient models based on ATR and DRIFTS spectra required spectra derivatization. Table S1 in the Supplementary Materials lists information regarding the regions and pretreatment methods applied during modeling.

In the case of bark samples, the obtained PLS prediction curves for salicylates were characterized by the correlation coefficient values 0.984–0.991. The lowest RSEP error values for TSA determination in the calibration/validation sets were found for the models built based on Raman and ATR spectra at 6.0/6.7% and 7.2/7.3%, respectively. For the DRIFTS technique, models based on MIR and NIR data of the bark samples were characterized by RSEP_val_ values that were in the 8.1–8.9% range. Internal validation of the models through cross-validation resulted in R_cv_ values of 0.891–0.949. Detailed parameters of the constructed PLS models are collected in Table 1. Prediction plots for the salicylates content modeling on the basis of ATR and Raman spectra are shown in Figure 6. These plots for DRIFTS and NIR data are presented in Figure S6 in the Supplementary Materials, while, in Figure S7, the RMSECV, variable importance in the projection (VIP) scores, and PLS regression vector plots are shown.

**Figure 7:**
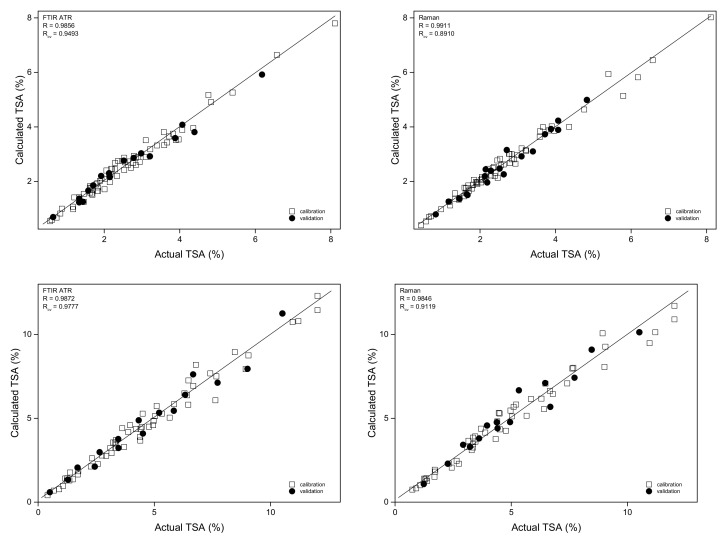
Prediction plots for the TSA content modeling in the bark (**top**) and leaves (**bottom**) on the basis of ATR (**left**) and Raman (**right**) spectra.

The PLS models obtained for TSA determination in the leaves were of comparable quality to those constructed for the bark samples (Figure 7 and Figure S6 in the Supplementary Materials), in spite of the fact that a higher content of TSA, up to 12% (*w*/*w*), was found in the leaf material.

A model utilizing NIR spectra had the best prediction ability, for which the calibration and validation set RSEP errors were in the 8.1–8.5% range. These errors for the models built using IR and Raman spectra were approximately 1 to 2% higher (Table 1). Compared to previously reported NIR analyses of poplar bark [5], our model was characterized by standard errors of calibration (SEC) and predictions (SEP) at least two times lower (Table S1 in the Supplementary Materials). Internal validation of the obtained calibration models for TSA quantification resulted in R_cv_ parameter values of 0.912–0.982. Figure S8 in the Supplementary Materials shows plots of the RMSECV, VIP scores, and regression vectors for all four techniques applied for TSA determination in poplar leaves. The leaves’ chemical composition complexity and the presence of additional compounds not present in bark, including proteins, carotenoids, and chlorophylls, could influence the accuracy of salicylates quantification in this sample type.

#### 2.3.2. Determination of Total Flavonoids (TFL)

The flavonoids content in the analyzed bark samples varied from 0.1 to 0.5% (*w*/*w*); in leaves, the flavonoid concentration was approximately three times higher and changed by 0.4–1.8% (*w*/*w*). No correlation between the TSA and TFL contents was observed for the bark samples (R^2^ = 0.06), but in the leaves, these two groups of chemical compounds displayed a weak correlation (R^2^ = 0.24).

Models built to determine the TFL content in poplar leaves were generally of higher quality than those constructed for bark material. The best calibration models based on IR data required spectra derivatization similar to that in TSA modeling. In leaves, five to seven factors were utilized to build PLS models. The prediction curves were characterized by correlation coefficients of 0.985–0.995, but the R_cv_ parameter for the developed models varied from 0.856 to 0.910. Quantification of TFL in the leaves resulted in RSEP errors of 3.4–6.0% and 4.5–6.6% for the calibration and validation sets, respectively (Table 1). The lowest RSEP_val_ values were obtained for the models based on NIR data, but the highest R_cv_ parameter was found for the model constructed utilizing Raman spectra. Figure S9 in the Supplementary Materials presents the prediction curves for the TFL content in leaf modeling based on Raman and IR data, and the RMSECV, VIP scores, and regression vectors are shown in Figure S10.

Quantification errors of the TFL content determination in the bark samples were slightly higher than those obtained for their analysis in the leaves, but as one could notice, the flavonoid content in the bark material was three times lower than it was in the leaves. The highest quality model for TFL was constructed utilizing Raman spectra, for which the RSEP errors for the calibration and validation sets were found to be 7.4 and 7.5%, respectively. The use of ATR and DRIFTS spectra in the MIR and NIR ranges for the bark samples resulted in calibration models of comparable quality characterized by RSEP_val_ values of 8.6–9.2% (Table 1). The prediction curves, the RMSECV plots, VIP scores, and regression vectors for the flavonoid content in the bark samples modeling based on Raman and IR data are shown in Figures S11 and S12 in the Supplementary Materials.

These models’ quality was closely related to very low concentrations of the analyzed compounds in the bark materials. It is worth noting that the variability of the polysaccharide content can mask flavonoid spectral contributions. Therefore, to improve the calibration model quality, the number of samples used for modeling should be noticeably increased.

### 2.4. Comparison of Vibrational Techniques

The obtained results show the extraordinary usefulness of the four applied vibrational spectroscopy techniques in the quantitative analysis of the active compounds, even in the case of the analysis of difficult plant materials. Nevertheless, it is necessary to remember that the quality of the obtained models depends on several factors, including the concentrations of the studied compounds, the presence of other substances, the quality of the spectra, and finally, the quality of the reference analyses. This last issue seems to be particularly important when extracting an analyzed substance from a complex matrix, such as plant tissue. Various protocols result in different extraction yields, and the determined content of the quantified compound varies from method to method. In the case of vibrational spectroscopy, the extracted and retained shares of the analyzed substance can be quantified.

IR and Raman techniques provide similar chemical information for the studied systems. In addition, elaborated protocols give results of comparable quality, but specific differences can be seen.

1. The Raman, ATR, and NIR spectra of plant materials can be recorded without any additional sample treatment, but collecting DRIFTS spectra in the MIR range requires diluting the sample in KBr.
2. A sample mass of a few milligrams is required to register the spectra. The same sample can be used applying various methods. On the other hand, repeated measurements for different portions of the same sample are required for heterogeneous materials.
3. Salicylates can be effectively quantified in poplar bark and leaves by using any of the applied spectroscopic techniques; the most robust model for bark was constructed based on Raman spectra, while, in the case of leaf materials, the lowest RSEP_val_ were found for NIR data. The flavonoids content in the leaves can be accurately determined through all four techniques, but for the bark samples, Raman spectroscopy gives the lowest errors of prediction.
4. Raman calibration models for TSA and TFL determination were developed using mean-centered spectra. In the case of IR data, better models were obtained based on the first or second spectra derivatives.
5. Raman spectra of the studied plant materials are characterized by a relatively low signal-to-noise ratio value. Therefore, to obtain reliable data, a longer data acquisition time is required compared to the IR techniques.
6. In the case of the Raman experiment, fluorescence can significantly affect the registered spectra.
7. Vibrational spectroscopy can be applied to quantify various groups of chemical compounds simultaneously, significantly accelerating and simplifying the analysis of the plant materials.

## 3. Materials and Methods

### 3.1. Plant Materials

The poplar (*Populus* L.) bark and leaf samples were collected from various species and habitats in Poland in 2018–2019. Immediately after harvesting, the tops of young shoots were separated into leaf and shoot groups, preliminarily shredded, and dried in the shade at room temperature for three weeks. After drying, 10-g portions of the plant material were homogenized with a laboratory mill and sieved through a 0.355-mm sieve. The prepared material was stored in a cool and dark place until extraction and a spectroscopic analysis. A set of 105 bark and 80 leaf samples was analyzed during the study.

### 3.2. Reference Analysis

The total salicylates (TSA) content in the studied plant material, calculated as salicin, was determined for the methanol–water extracts after hydrolysis using the ultra-high performance liquid chromatographic method coupled with ultraviolet detection (UHPLC–UV) [49]. Briefly, 250.0 mg of the sieved sample were mixed with 20 mL NaOH (0.11 M) in aqueous MeOH solution (50% *v*/*v*) and placed in a water bath (60 °C); the extraction process and alkaline hydrolysis took one hour. Next, the samples were cooled and minimally acidified by adding 1.0 mL HCl (2.86 M), then adjusted to 25.0 mL. After centrifugation of about 2 mL of the hydrolyzed extract, the supernatant was filtered (0.22-µm PVDF membrane), and the final extract with a 1:100 (*w/v*) drug-to-solvent ratio (DSR) was stored in the dark at 4° C until the analysis. Separately, a seven-point calibration curve for salicin in MeOH was prepared over a primary concentration range of 0.015–1.0 mg/mL. Chromatographic separation was performed with the UHPLC-DAD Dionex Ultimate 3000 System on a modified reversed stationary phase (Kinetex F5 column, 150 × 2.1 mm, grain diameter 2.6 μm) using a mobile phase composed of 0.1% HCOOH (*v*/*v*) in water (A) and 0.1% HCOOH (*v*/*v*) in methanol (B), with the following gradient: 0→7 min, 0→9% B in A, then 7–7.5 min, 9→95% B in A 0.4 mL/min at 35 °C. The injection volume for the samples and standards was 2 µL. Under these conditions, salicin was eluted at 6.1 min. The concentration of total salicinoids expressed as salicin was determined by measuring the absorbance at 269 nm.

The total content of flavonoids (TFL), expressed as quercetin, was determined spectrophotometrically [50]. Ultrasonically assisted extraction of the 50.0-mg sieved sample with 10.0 mL aqueous MeOH 50% (*v*/*v*) was performed for a period of 15 min at room temperature using a thermostated ultrasonic bath at 50% of its power (Sonorex 10P; Bandelin, Berlin, Germany). After centrifugation, the supernatant was collected and immediately analyzed. Quercetin standards in the concentration range of 3–250 μg/mL in 50% MeOH (aq.) were prepared. A volume of 125 µL of AlCl_3_ (0.4%) solution in aqueous MeOH solution (50% *v*/*v*) and 25 µL of the quercetin standard, plant extract, or blank sample (aqueous MeOH solution (50% *v*/*v*) were poured into the wells of a 96-well flat-bottomed plate. The flavonoid concentration was determined by measuring the absorbance at 420 nm 90 min after mixing the sample with the complexing reagent.

### 3.3. Chemicals and Reagents

All chemical reagents used in the study were of analytical grade and were obtained from Merck (Darmstadt, Germany; HPLC solvents) or from POCh (Lublin, Poland; MeOH, NaOH, HCl, and AlCl_3_). The standards of quercetin (≥99%) and salicin (≥99%) were purchased from CarlRoth (Karlsruhe, Germany) and Sigma-Aldrich (Steinheim, Germany), respectively.

### 3.4. Spectroscopic Conditions

IR and NIR spectra of the powdered poplar material were recorded using an iS50 FTIR spectrometer (Thermo Nicolet, Madison, WI, USA). Interferograms were averaged over 128 scans, which were then Happ-Genzel apodised and Fourier-transformed to give spectra in the 400–4000 cm^−1^ and 4000–9000 cm^−1^ ranges with a resolution of 4 cm^−1^. An average spectrum used for modeling was computed from three independent measurements for each sample. ATR-FTIR spectra were recorded using a single reflection Golden Gate (Specac, Slough, UK) diamond accessory; a KBr beam splitter and DTGS detector were used for the measurements.

Diffuse–reflectance spectra were registered in Kubelka-Munk units using a Seagull (Harrick Scientific, Pleasantville, NY, USA) optical assembly mounted in the iS50 unit. DRIFTS/NIR spectra were obtained utilizing a CaF_2_ beam splitter and DTGS detector. To record the spectra of plant materials diluted with a dried KBr in the MIR range, a KBr beam splitter and DTGS detector were applied.

Raman spectra of samples in the form of pellets, obtained by pressing 150 mg of powdered plant material and applying a force of 5 T/cm^2^, were registered using an iS50 FTIR spectrometer with a Raman accessory (Thermo Nicolet, Madison, WI, USA). A CaF_2_ beamsplitter and indium–gallium–arsenide detector were utilized. The samples were illuminated by a Nd:YVO_4_ laser (λ = 1.064 µm), with a power of 100 mW at the sample. Backscattered radiation was collected from 16 points of the pellet surface. Interferograms were averaged over 256 scans; then, they were Happ-Genzel apodised and Fourier-transformed to give the spectra in the 100–3700 cm^−1^ range with a resolution of 8 cm^−1^.

### 3.5. Software and Numerical Data Treatment

Principal component analysis (PCA) of the spectral data was performed by applying the PLS-Toolbox (Eigenvector, Wenatchee, WA, USA) in the MATLAB environment (MathWorks, Natick, MA, USA). Partial least squares (PLS) regression models were built utilizing TQ Analyst software (Thermo Fisher, Madison, WI, USA). Spectra were normalized using the MSC procedure [51]; in the case of ATR and DRIFTS data, the first and second derivatives of IR spectra were calculated, respectively, using Savitzky-Golay smoothing [52] and applying 23 data points and a third-order polynomial. For the PCA and PLS modeling purposes, the spectral data were mean-centered. Applying the PCA score plots, 75% of the samples were randomly selected for calibration, and 25% of the remaining were used for test set validation of the models. An internal validation was performed using the cross-validation (leave-*n*-out, *n* = 2) procedure, resulting in the R_cv_ parameter value. A root mean square error of cross-validation (RMSECV) was calculated to establish an optimal number of factors for PLS modeling [53].

To compare the predictive abilities of the constructed models, the relative standard error of prediction (RSEP) values for the calibration and validation sets were calculated according to the following equation:

(1) $$\mathrm{RSEP}\left(\%\right)=\sqrt{\frac{\sum_{i=1}^{n}{\left(C_{i}-C_{i}^{A}\right)}^{2}}{\sum_{i=1}^{n}C_{i}^{A}{}^{2}}}\times 100,$$

where *C^A^* is a compound content determined by the reference method, *C* is the value found from PLS modeling, and *n* is the number of samples.

## 4. Conclusions

A comprehensive comparison was made between Raman spectroscopy, diffuse reflectance in the near- and mid-infrared regions, and attenuated total reflection utilized for the quantification of salicylates and flavonoids in poplar bark and leaves. The obtained results indicate that vibrational spectroscopy supported by multivariate modeling can be an effective tool for the direct quantitative determination of active compounds in plant materials. The lowest RSEP errors of the order of 6.0–7.5% for active component determination in poplar bark were found using Raman spectroscopy. In the cases of the TSA and TFL analyses on poplar leaves, the lowest RSEP errors in the 3.4–8.5% range were obtained when applying NIR spectra. The presented analyses were performed for the powdered samples without additional chemical treatment. The developed procedures can find application in the fast and simultaneous quantifying of different groups of active compounds in dried plant materials, effectively supporting raw material analyses in the food, cosmetic, and pharmaceutical industries.

## Figures and Tables

**Table 1:** Calibration parameters of the TSA and TFL content modeling in the poplar samples.

Plant Material	Active Compound	Technique	Preprocessing	R_cal_	R_val_	RSEP_cal_	RSEP_val_	R_cv_	LV
	TSA	ATR	2nd der	0.989	0.986	7.17	7.25	0.949	6
	TSA	DRIFTS/MIR	2nd der	0.989	0.979	6.88	8.09	0.928	5
	TSA	NIR	MSC	0.984	0.972	8.01	8.85	0.948	8
	TSA	RAMAN	none	0.991	0.983	6.03	6.67	0.891	10
bark									
	TFL	ATR	2nd der	0.985	0.971	6.99	9.14	0.891	6
	TFL	DRIFTS/MIR	1st der	0.973	0.972	9.17	9.18	0.834	7
	TFL	NIR	MSC	0.979	0.965	8.25	8.64	0.838	7
	TFL	RAMAN	none	0.982	0.983	7.35	7.45	0.873	8
	TSA	ATR	2nd der	0.987	0.983	8.48	9.20	0.977	5
	TSA	DRIFTS/MIR	1st der	0.987	0.980	8.36	9.56	0.963	4
	TSA	NIR	1st der	0.986	0.989	8.53	8.07	0.982	5
	TSA	RAMAN	none	0.985	0.975	9.26	9.95	0.912	8
leaves									
	TFL	ATR	2nd der	0.988	0.978	4.79	5.20	0.885	7
	TFL	DRIFTS/MIR	1st der	0.985	0.975	5.98	5.69	0.857	6
	TFL	NIR	1st der	0.995	0.983	3.40	4.47	0.856	5
	TFL	RAMAN	none	0.986	0.973	5.48	6.59	0.910	6

TSA—total salicylates in 50% MeOH extract (calculated as salicin); TFL—total flavonoids in 50% MeOH extract (calculated as quercetin); MSC—Multiplicative Scatter Correction; der—derivative; RSEP—Relative Standard Error of Prediction; LV—Latent Variable.

## Data Availability

Not applicable.

## Supplementary Materials

The following supporting information can be downloaded at https://www.mdpi.com/article/10.3390/molecules27123954/s1: Table S1: Calibration parameters and spectral ranges used for TSA and TFL content modeling. Figure S1: ATR spectra of poplar leaves and bark containing extreme contents of salicylates, difference spectra, and the spectrum of salicin. Figure S2: PCA loadings for poplar bark and leaves on the basis of DRIFTS and NIR spectra. Figure S3: PCA for poplar bark samples on the basis of uncorrected Raman spectra; scores plots and average spectra of the highlighted groups of objects. Figure S4: Average Raman spectrum of poplar leaves together with β-carotene and salicin spectra. Figure S5: PC1/PC2 and PC1/PC3 score plots obtained based on ATR spectra of leaves with the TSA content given. Figure S6: Prediction plots for TSA content modeling in the bark and leaves on the basis of DRIFTS and NIR spectra. Figure S7: VIP scores and regression coefficient plots for PLS modeling of the TSA content in poplar leaves on the basis of ATR, DRIFTS, NIR, and Raman spectra. Figure S8: VIP scores, regression coefficients, and RMSECV plots for PLS modeling of the TSA content in poplar leaves on the basis of ATR, DRIFTS, NIR, and Raman spectra. Figure S9: Prediction plots for the TFL content modeling in leaves on the basis of ATR, DRIFTS, NIR, and Raman spectra. Figure S10. VIP scores, regression coefficients, and RMSECV plots for PLS modeling of the TFL content in poplar leaves on the basis of ATR, DRIFTS, NIR, and Raman spectra. Figure S11: Prediction plots for TFL content modeling in poplar bark on the basis of ATR, DRIFTS, NIR, and Raman spectra. Figure S12: VIP scores, regression coefficients, and RMSECV plots for PLS modeling of the TFL content in poplar bark on the basis of ATR, DRIFTS, NIR, and Raman spectra.
