# Supplementary material for: Quantification of Salicylates and Flavonoids in Poplar Bark and Leaves Based on IR, NIR, and Raman Spectra
Source: Molecules. 2022 Jun 20;27(12):3954. doi: 10.3390/molecules27123954 (PMC9229158; doi:10.3390/molecules27123954)
Supplement: Supplementary file 1 [file molecules-27-03954-s001.zip › molecules-1757377-supplementary.pdf]

Quantification of salicylates and flavonoids in poplar bark and leaves based on IR, NIR, and Raman spectra

Sylwester Mazurek[a*], Maciej Włodarczyk[b*], Sonia Pielorz[a], Piotr Okińczyc[b], Piotr M. Kuś[b], Gabriela Długosz[b], Diana Vidal-Yañez[b,c], Roman Szostak[a]

[a] Department of Chemistry, University of Wrocław, 14 F. Joliot-Curie, 50-383 Wrocław, Poland

[b] Department of Pharmacognosy and Herbal Medicines, Faculty of Pharmacy, Wroclaw Medical University, 211a Borowska, 50-556 Wrocław, Poland

[c] University of Barcelona, Faculty of Pharmacy, Joan XXIII, 27-31, 08014 Barcelona, Spain, Spain

E-mail addresses: sylwester.mazurek@chem.uni.wroc.pl (S.Mazurek), maciej.wlodarczyk@umw.edu.pl (M. Włodarczyk)

Supplementary Material

List of content:

Table S1. Calibration parameters and spectral ranges used for TSA and TFL content modeling.
Figure S1. ATR spectra of poplar leaves (left) and bark (right) containing extreme content of salicylates, difference spectra and spectrum of salicin.
Figure S2. PCA loadings for poplar bark (left panel) and leaves (right panel) on the basis of DRIFTS (top) and NIR (bottom) spectra.
Figure S3. PCA for poplar bark samples on the basis of uncorrected Raman spectra; scores plots (top) and average spectra of the highlighted groups of objects (bottom).
Figure S4. Average Raman spectrum of poplar leaves together with β-carotene and salicin spectra.
Figure S5. PC1/PC2 (left) and PC1/PC3 (right) score plots obtained based on ATR spectra of leaves with TSA content given.
Figure S6. Prediction plots for TSA content modeling in bark (top panel) and leaves (bottom) on the basis of DRIFTS (left) and NIR (right) spectra.
Figure S7. VIP scores and regression coefficient plots for PLS modeling of TSA content in poplar leaves on the basis of ATR, DRIFTS, NIR and Raman spectra (from top to bottom).
Figure S8. VIP scores, regression coefficients and RMSECV plots for PLS modeling of TSA content in poplar leaves on the basis of ATR, DRIFTS, NIR and Raman spectra (from top to bottom).
Figure S9. Prediction plots for TFL content modeling in leaves on the basis of ATR, DRIFTS, NIR and Raman spectra.
Figure S10. VIP scores, regression coefficients and RMSECV plots for PLS modeling of TFL content in poplar leaves on the basis of ATR, DRIFTS, NIR and Raman spectra (from top to bottom).
Figure S11. Prediction plots for TFL content modeling in poplar bark on the basis of ATR, DRIFTS, NIR and Raman spectra.
Figure S12. VIP scores, regression coefficients and RMSECV plots for PLS modeling of TFL content in poplar bark on the basis of ATR, DRIFTS, NIR and Raman spectra (from top to bottom).

Table S1. Calibration parameters and spectral ranges used for TSA and TFL content modeling.

Plant material	Compound	Technique	Spectral range [cm^{-1}]	RMSEC	RMSEV	SEC	SEP	RMSECV
Bark	TSA	ATR	740-800<br>910-1700	0.207	0.209	0.208	0.225	0.390
Bark	TSA	DRIFT	910-1700	0.202	0.234	0.204	0.251	0.506
Bark	TSA	NIR	4510-4720<br>5360-7100	0.231	0.261	0.233	0.237	0.414
Bark	TSA	RAMAN	970-1750<br>2780-3200	0.171	0.191	0.178	0.197	0.605
Bark	TFL	ATR	850-1200<br>1340-1400<br>1500-1700<br>2800-3100	0.0174	0.0218	0.0178	0.0226	0.0473
Bark	TFL	DRIFT	1200-1350<br>1500-1800<br>3000-3700	0.0248	0.0242	0.0251	0.0264	0.0590
Bark	TFL	NIR	4150-4370<br>4500-7200	0.0209	0.0210	0.0210	0.0231	0.0566
Bark	TFL	Raman	340-480<br>1180-1750<br>2800-3150	0.0203	0.0203	0.0201	0.0220	0.0525
Leaves	TSA	ATR	1180-1800	0.458	0.505	0.463	0.525	0.615
Leaves	TSA	DRIFT	800-1800	0.456	0.541	0.459	0.572	0.788
Leaves	TSA	NIR	4500-4720<br>+ 5400-6500	0.464	0.483	0.469	0.513	0.516
Leaves	TSA	RAMAN	415-890<br>+ 1410-1490<br>+ 1570-1770<br>+ 3000-3290	0.521	0.559	0.526	0.653	1.210
Leaves	TFL	ATR	1480-1800<br>3100-3700	0.0448	0.0465	0.0453	0.0518	0.136
Leaves	TFL	DRIFT	1190-1530<br>1570-1860<br>3100-3700	0.0587	0.0537	0.0590	0.0568	0.177
Leaves	TFL	NIR	4030-4290<br>4500-4980	0.0326	0.0396	0.0330	0.0411	0.172
Leaves	TFL	Raman	270-463<br>1250-1700<br>2700-3100	0.0521	0.0583	0.0527	0.0776	0.131

* TSA – total salicilates in 50% MeOH extract (calculated as salicin)
** TFL - total flavonoids in 50% MeOH extract (calculated as quercetin)

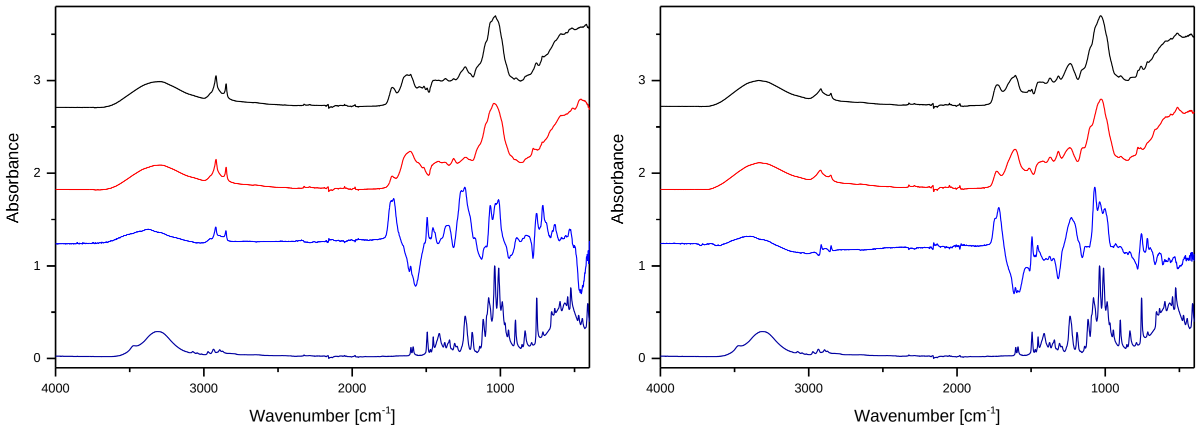

Figure S1. ATR spectra of poplar leaves (left) and bark (right) containing extreme content of salicylates, difference spectra and spectrum of salicin.

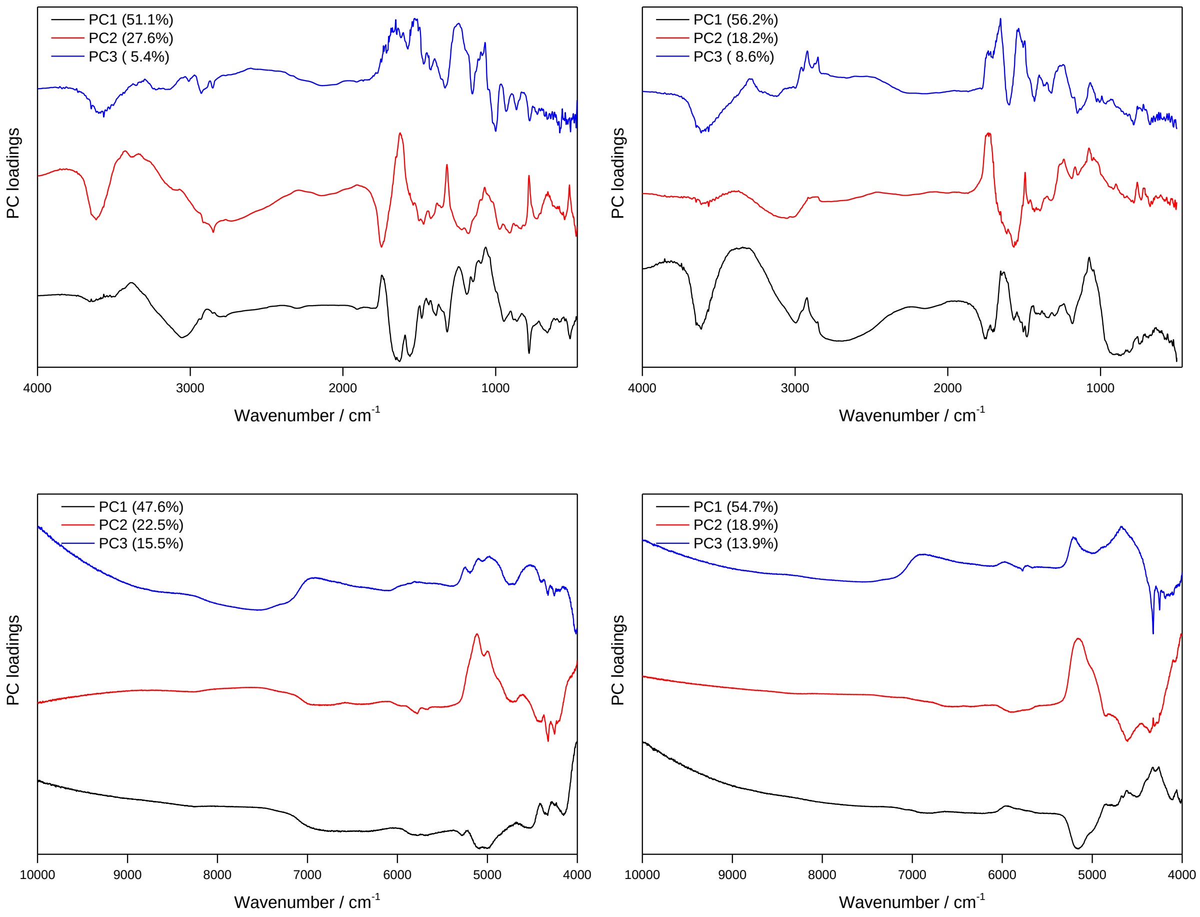

Figure S2. PCA loadings for poplar bark (left panel) and leaves (right panel) on the basis of DRIFTS (top) and NIR (bottom) spectra.

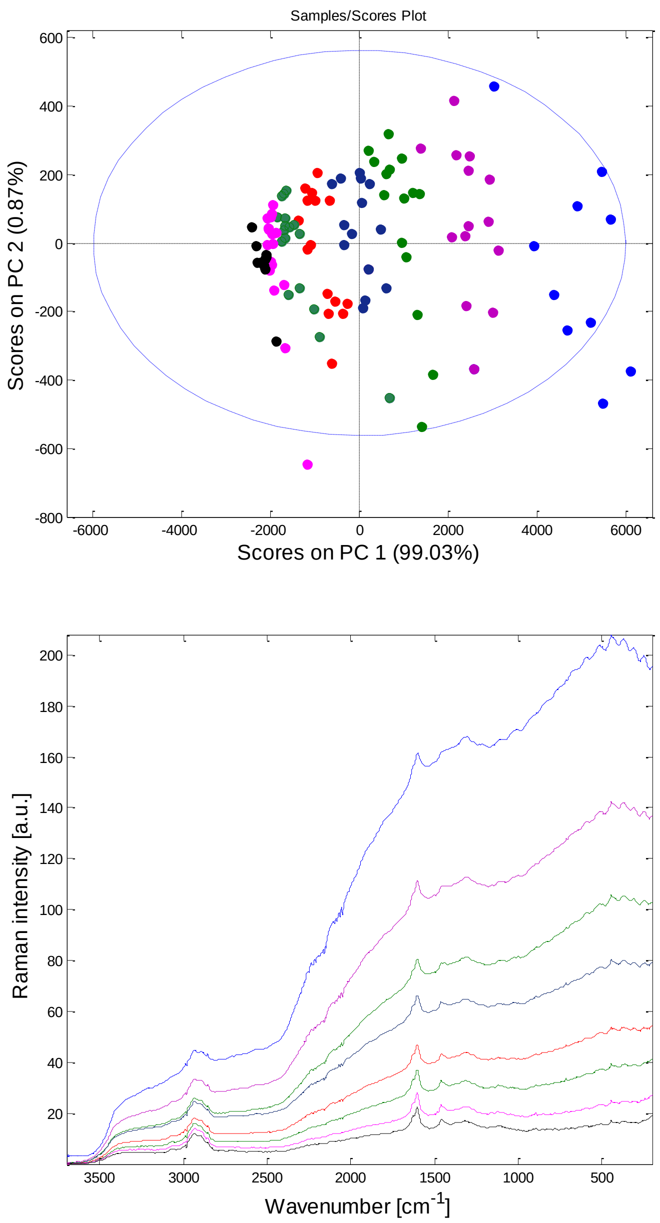

Figure S3. PCA for poplar bark samples on the basis of uncorrected Raman spectra; scores plots (top) and average spectra of the highlighted groups of objects (bottom).

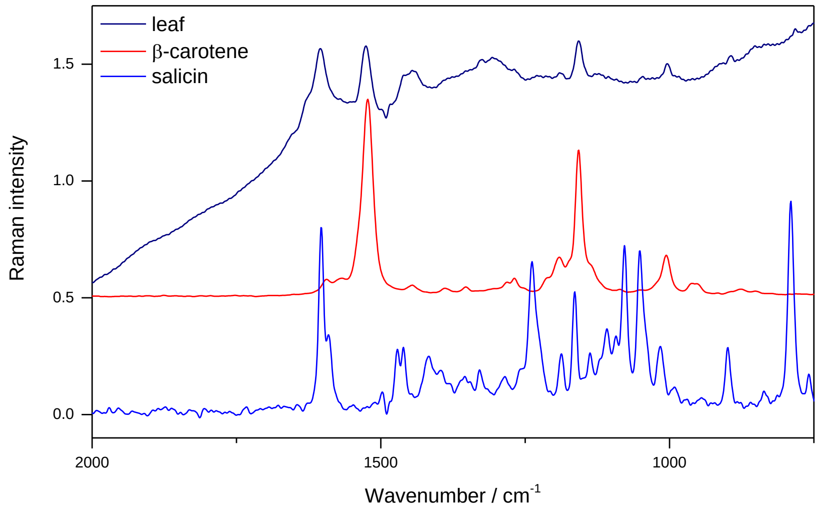

Figure S4. Average Raman spectrum of poplar leaves together with β-carotene and salicin spectra

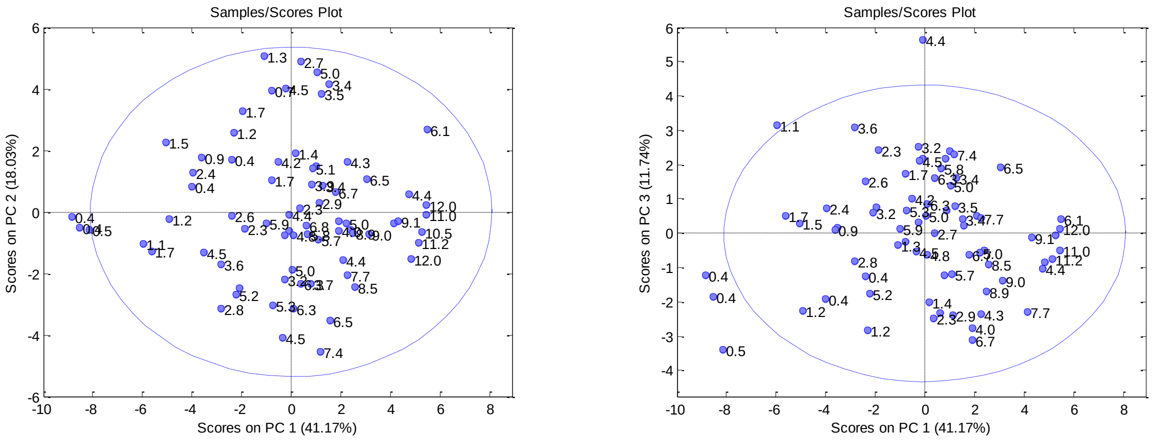

Figure S5. PC1/PC2 (left) and PC1/PC3 (right) score plots obtained based on ATR spectra of leaves with TSA content given.

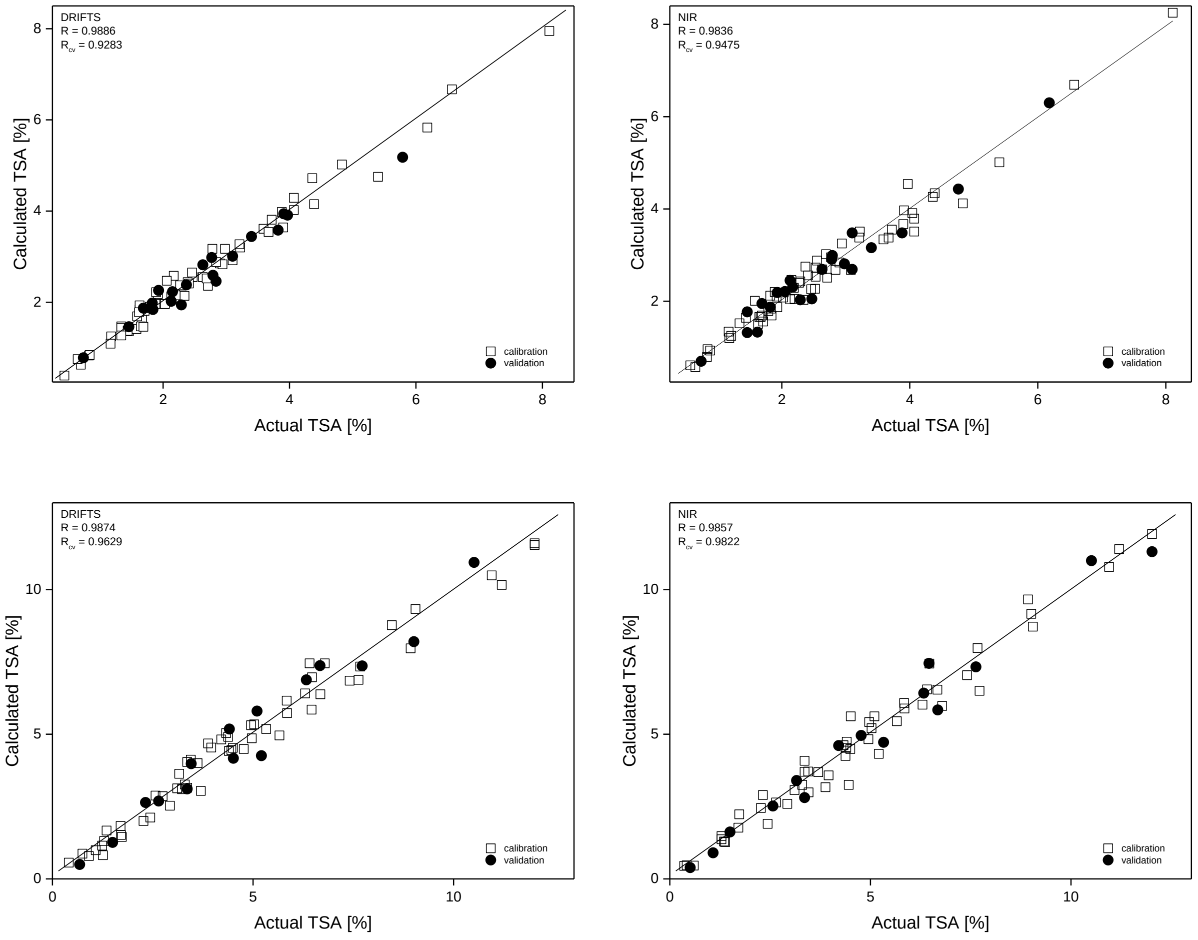

Figure S6. Prediction plots for TSA content modeling in bark (top panel) and leaves (bottom) on the basis of DRIFTS (left) and NIR (right) spectra.

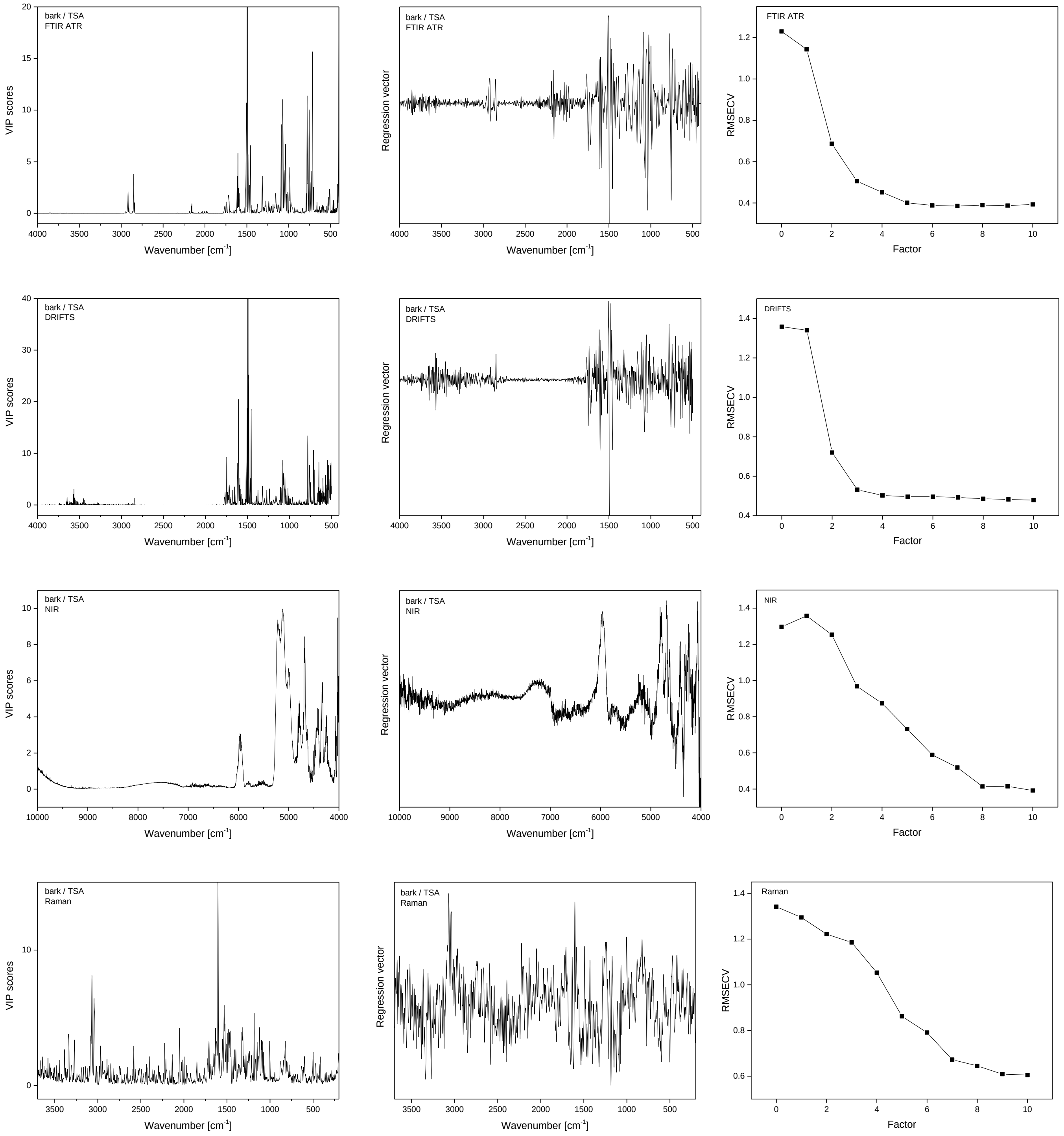

Figure S7. VIP scores and regression coefficient plots for PLS modeling of TSA content in poplar leaves on the basis of ATR, DRIFTS, NIR and Raman spectra (from top to bottom).

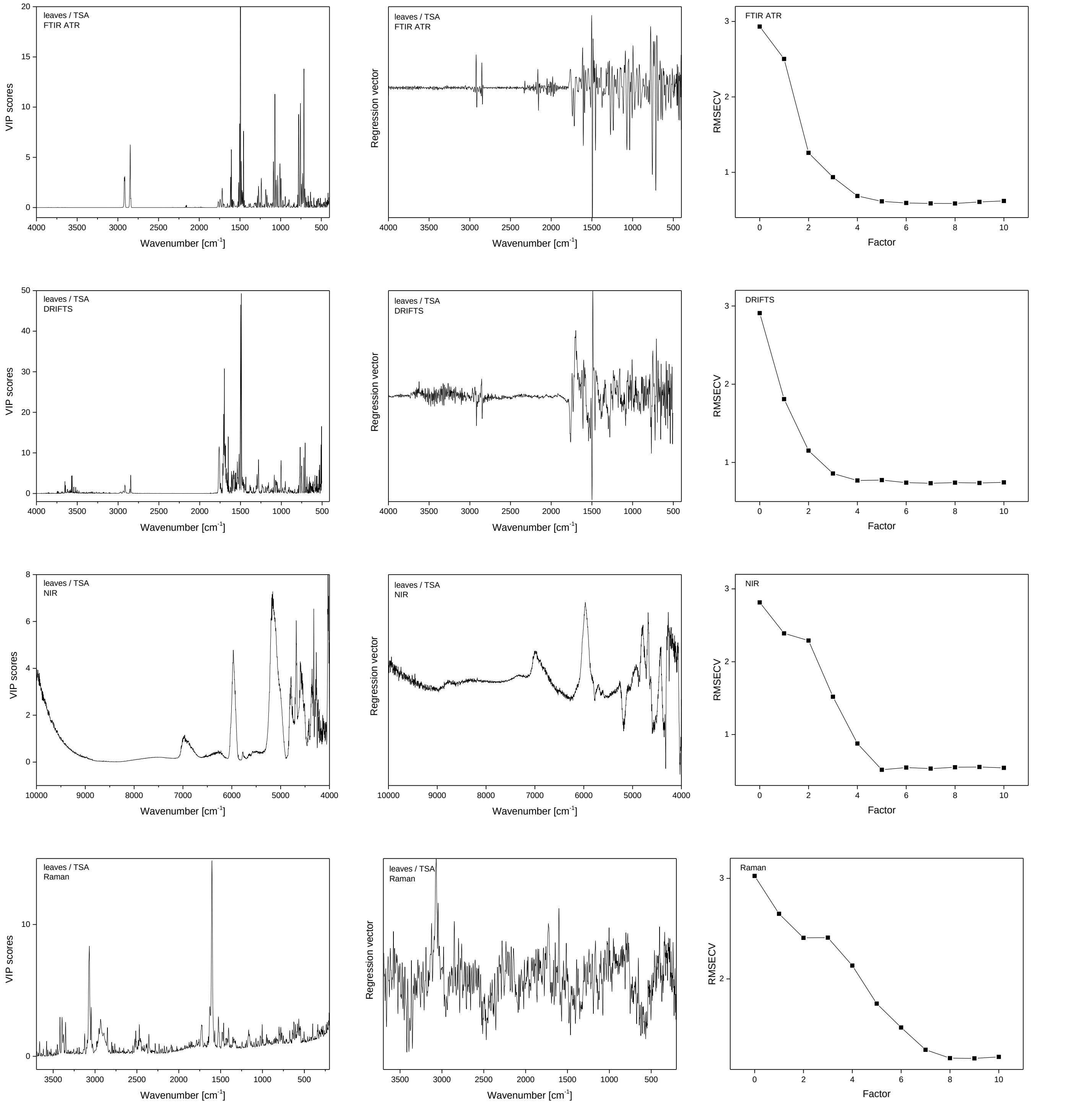

Figure S8. VIP scores, regression coefficients and RMSECV plots for PLS modeling of TSA content in poplar leaves on the basis of ATR, DRIFTS, NIR and Raman spectra (from top to bottom).

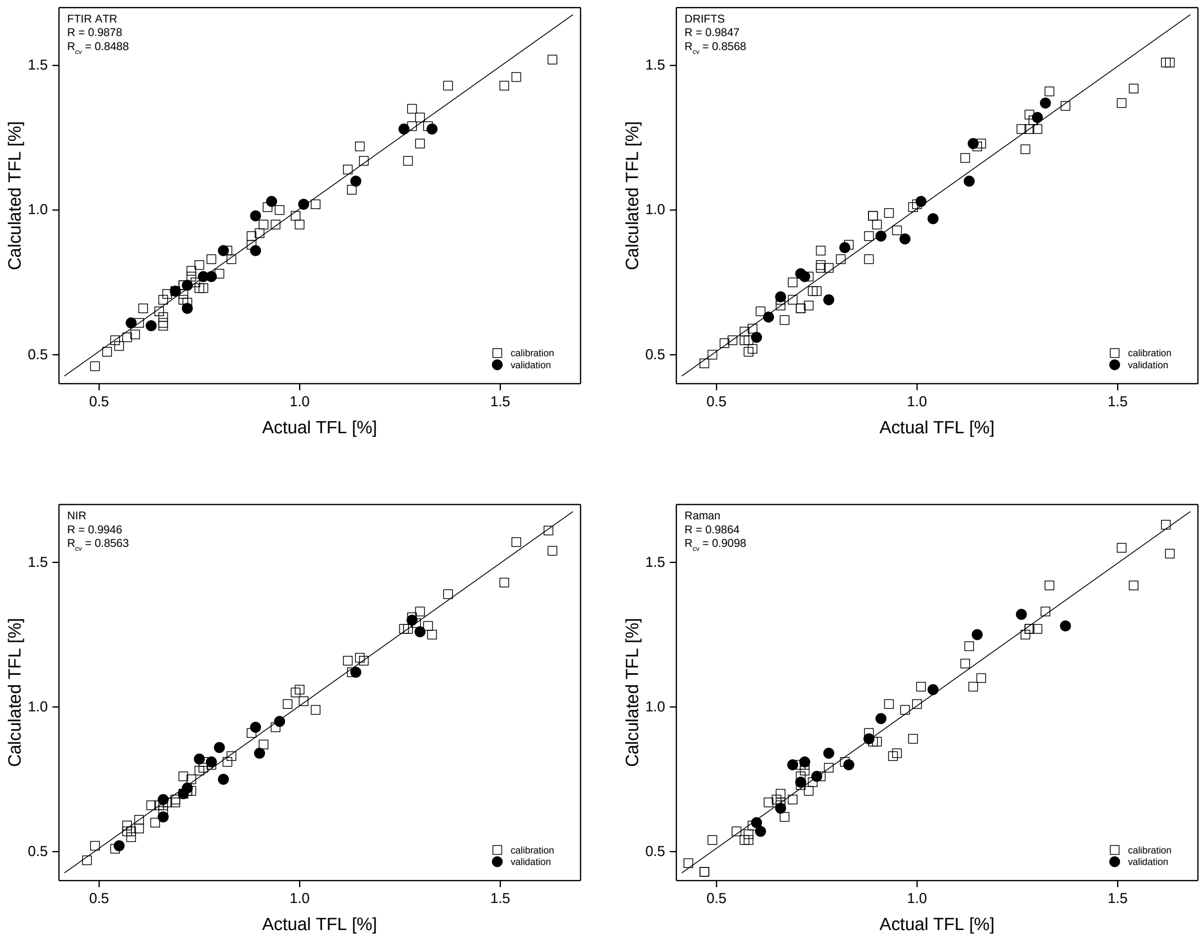

Figure S9. Prediction plots for TFL content modeling in leaves on the basis of ATR, DRIFTS, NIR and Raman spectra.

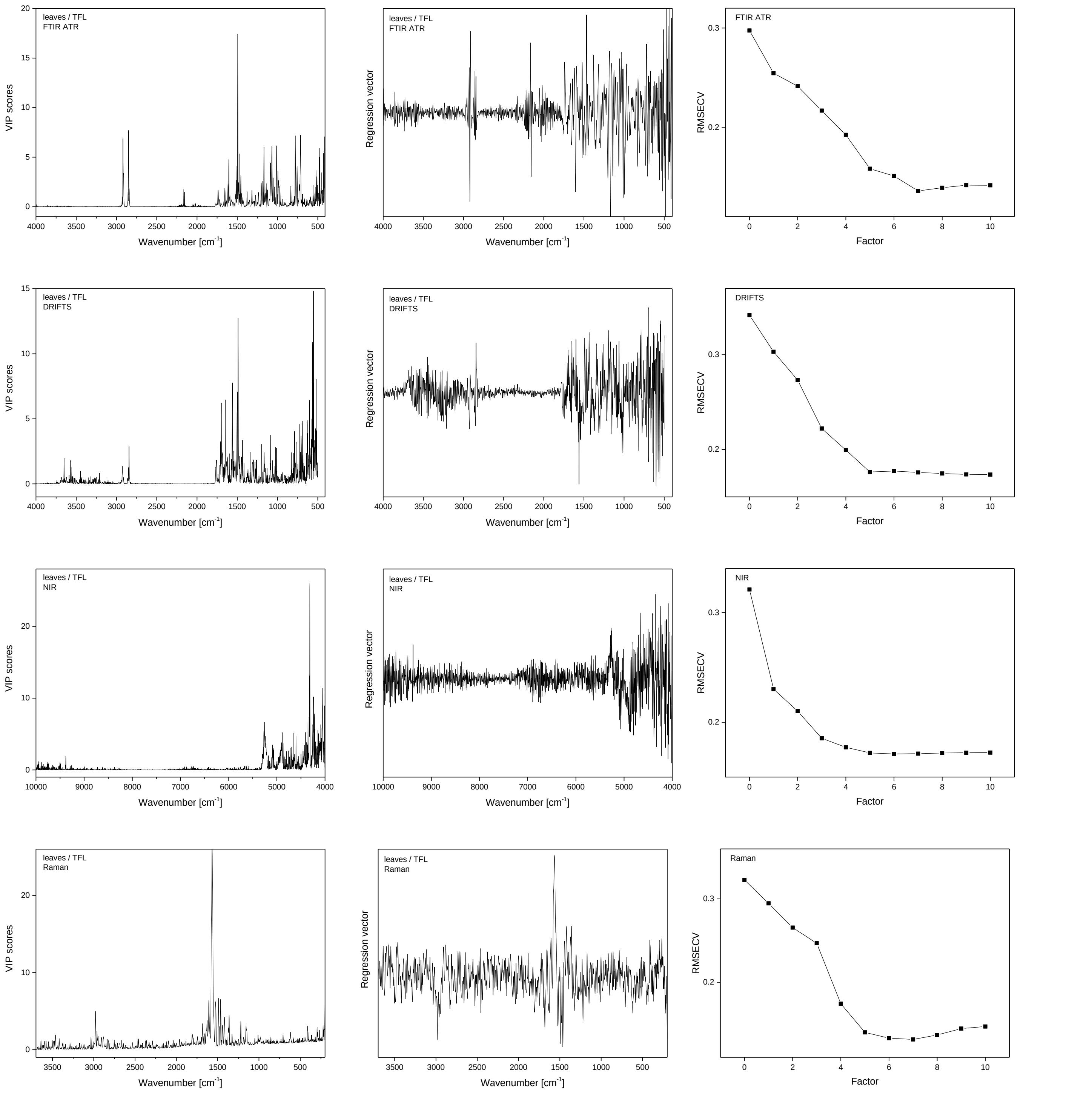

Figure S10. VIP scores, regression coefficients and RMSECV plots for PLS modeling of TFL content in poplar leaves on the basis of ATR, DRIFTS, NIR and Raman spectra (from top to bottom).

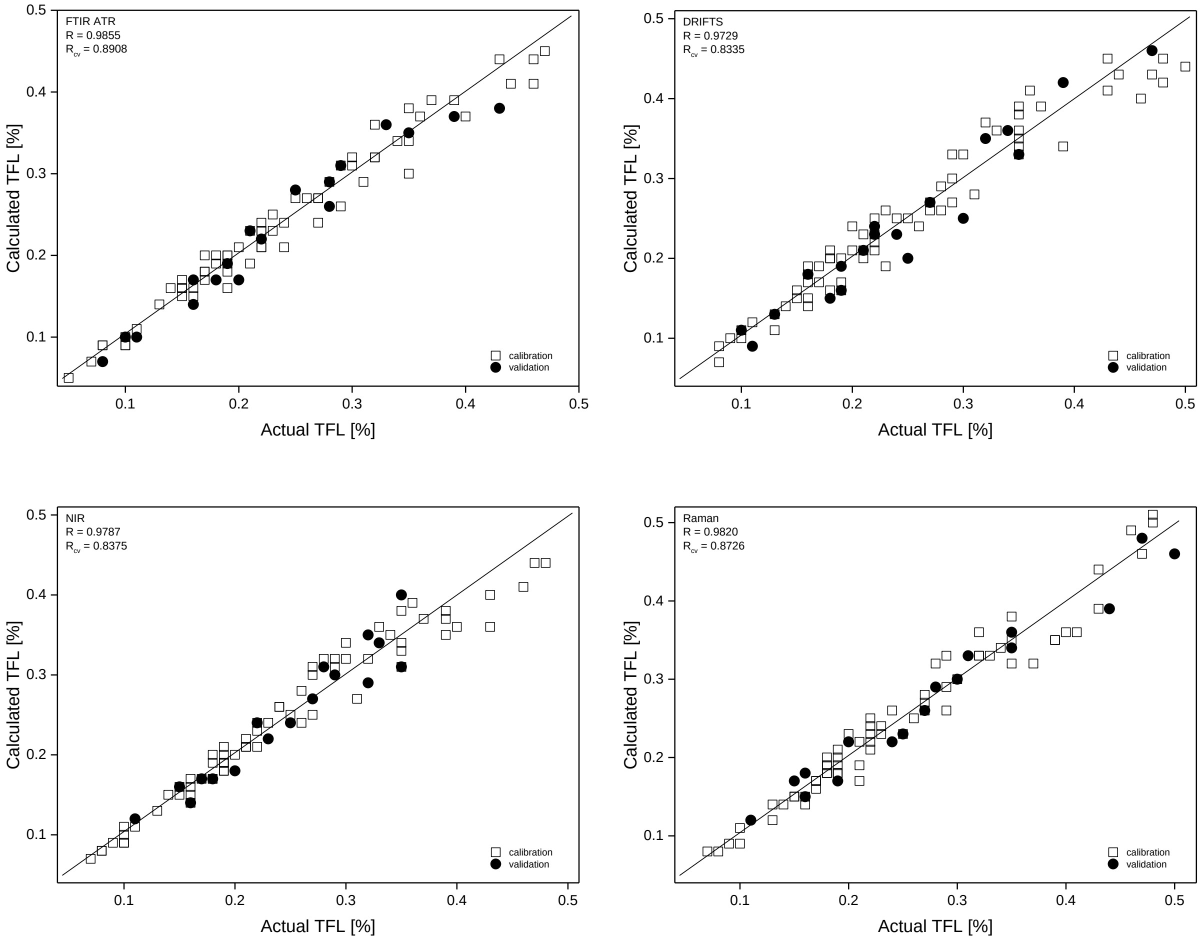

Figure S11. Prediction plots for TFL content modeling in poplar bark on the basis of ATR, DRIFTS, NIR and Raman spectra.

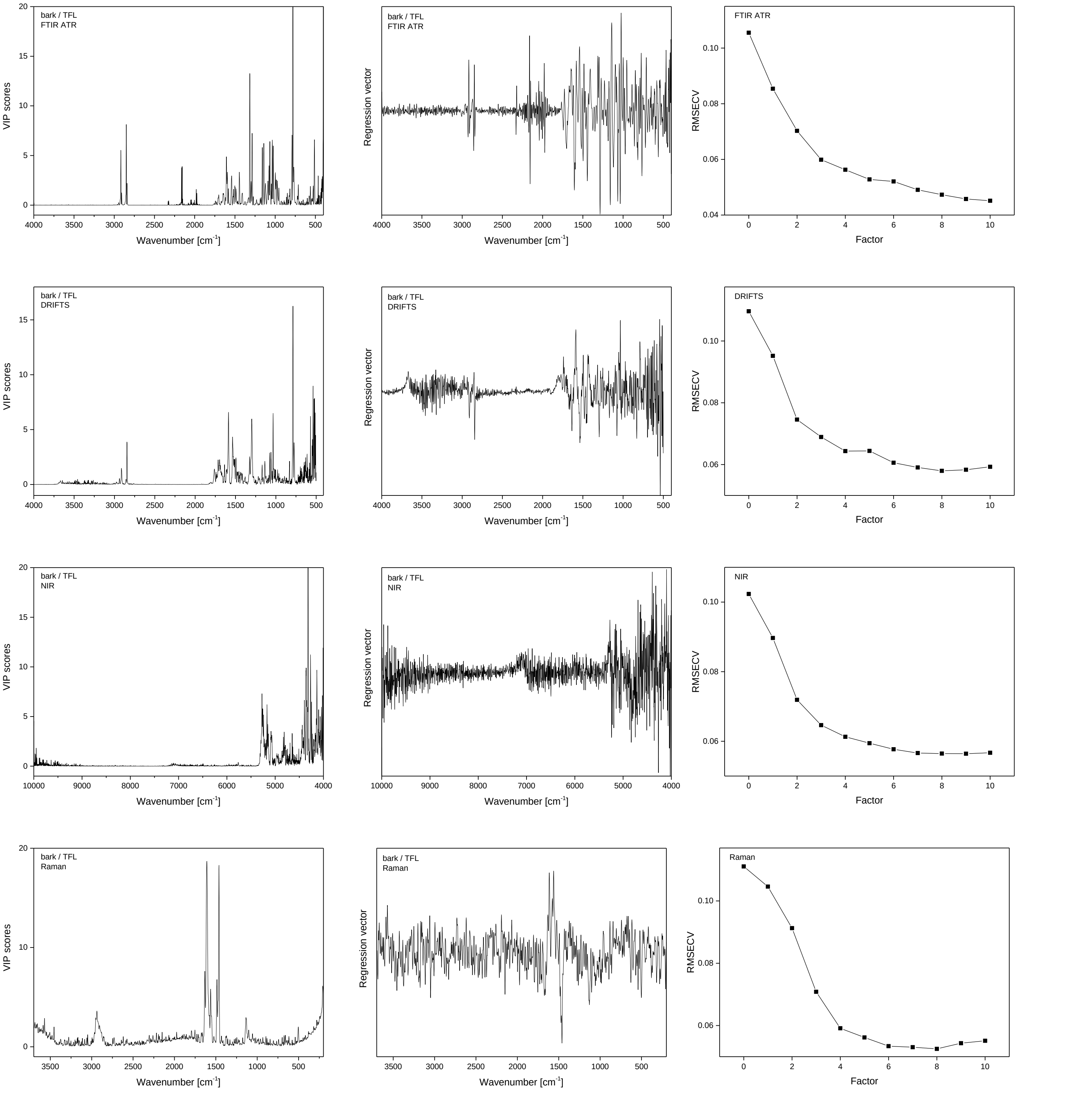

Figure S12. VIP scores, regression coefficients and RMSECV plots for PLS modeling of TFL content in poplar bark on the basis of ATR, DRIFTS, NIR and Raman spectra (from top to bottom).
